# Clarification of Reported Statistical Inconsistencies in a CHA2DS2‐VA Analysis of No‐Reflow After Primary Percutaneous Coronary Intervention

**DOI:** 10.1002/clc.70478

**Published:** 2026-10-03

**Authors:** Fatih Akkaya, Nihan Bahadır

**Affiliations:** ^1^ Department of Cardiology, Faculty of Medicine Ordu University Ordu Türkiye

Dear Editor,

Sun et al. report that the CHA2DS2‐VA score predicts angiographic no‐reflow after primary percutaneous coronary intervention in women but not in men [1]. The hypothesis is clinically relevant, and the reported sex interaction is noteworthy. Three issues affecting interpretation, together with several reporting errors, require clarification.

First, Table 6 reports the number of stents implanted in men as an adjusted odds ratio of 1.844 (95% confidence interval [CI], 1.055–3.223) with *p* = 0.320. Because the CI excludes unity, the reported odds ratio and CI are consistent with *p* approximately 0.032, not 0.320; the univariable estimate is also significant (odds ratio, 1.899; 95% CI, 1.114–3.238; *p* = 0.018). Yet the Results state that only platelet count was an independent predictor in men, consistent with a nonsignificant stent effect. Thus, the adjusted *p* value, CI, and textual model interpretation are mutually inconsistent; the authors should clarify which is correct.

Second, Tables 5 and 6 report identical odds ratios, CIs, and *p* values for total stent length and number of lesion vessels within each sex‐stratified univariable analysis: 1.013 (95% CI, 0.986–1.041; *p* = 0.343) in women and 1.015 (95% CI, 0.996–1.034; *p* = 0.117) in men. In the overall cohort, these predictors differ (Table 4: odds ratios, 1.014 and 0.929, respectively). Repetition across two differently scaled predictors in both sex strata suggests a possible transcription or output‐transfer error. Verification against the original model output and correction of the affected rows are needed.

Third, the receiver operating characteristic analyses report sensitivity and specificity without the selected cutoffs, preventing reproduction or clinical application of the operating points. At the observed prevalence of 18.3% in women, the reported rounded sensitivity of 78.1% and specificity of 47.6% imply a positive predictive value of approximately 25%. Because the authors conclude that the score alone provides adequate risk stratification in women, the selected cutoff and corresponding positive and negative predictive values, with CIs, should be reported.

Several additional reporting errors warrant correction. In Table 3, CAD in the normal‐flow group is reported as 7 (11.1%), although the percentage, cohort totals, and reported *p* = 0.613 indicate that the intended count is 71; *p* values shown as “0.000” and the neutrophil‐to‐lymphocyte ratio entry “6.42 (583)” also require correction or confirmation.

We raise these points because the reported sex interaction merits further study, not to dismiss the hypothesis. Several table entries remain internally inconsistent, and the absent receiver operating characteristic thresholds limit clinical interpretation. Corrected tables and clarification of the original model output would allow readers to assess the robustness and applicability of the findings.

## Funding

The authors have nothing to report.

## Conflicts of Interest

The authors declare no conflicts of interest.

## Data Availability

Data sharing is not applicable to this article as no data sets were generated or analyzed during the current study.
